# Acute malnutrition among children aged 6–59 months of the nomadic population in Hadaleala district, Afar region, northeast Ethiopia

**DOI:** 10.1186/s13052-018-0457-1

**Published:** 2018-02-07

**Authors:** Zemichael Gizaw, Wondwoson Woldu, Bikes Destaw Bitew

**Affiliations:** 1 Department of Environmental and Occupational Health and Safety, University of Gondar, Gondar, Ethiopia; 2 Hadaleala District Health Office, Hadaleala District, Afar Regional State, Ethiopia

**Keywords:** Acute malnutrition, Children aged 6–59 months, Nomads, Afar region, Ethiopia

## Abstract

**Background:**

Acute malnutrition to be a major health burden in the world, particularly in the developing world. Acute malnutrition is associated with more than one third of the global disease burden for children. Malnourished children are physically, emotionally and intellectually less productive and suffer more from chronic illnesses and disabilities. The nature, magnitude and determinants of acute malnutrition are determined among the general populations; however, there is a lack of evidence in the nomadic communities.

**Methods:**

A cross-sectional study was conducted to assess the magnitude and factors associated with acute malnutrition among children aged 6–59 months in Hadaleala district, Afar Region. A total of 591 under-five children were included in this study, and subjects were recruited by the multistage cluster sampling technique. Data were collected by a pre-tested questionnaire and a simple anthropometric index so called mid-upper arm circumference (MUAC). The multivariable binary logistic regression analysis was used to identify factors associated with acute malnutrition on the basis of adjusted odds ratio (AOR) with 95% confidence interval (CI) and *p* < 0.05.

**Results:**

The prevalence of acute malnutrition was 11.8% (95% CI = 9.3, 14.8%). The highest prevalence (50%) of acute malnutrition occurred among children aged between 12.0–23.0 months. Childhood acute malnutrition was associated with the presence of two (AOR = 2.49, *p* < 0.05) and three (AOR = 12.87, *p* < 0.001) children in each household, unprotected drinking water sources (AOR = 3.78, *p* < 0.05), absence of the latrine (AOR = 5.24, *p* < 0.05), hand washing with soap (AOR = 0.21, *p* < 0.05), childhood diarrheal disease (AOR = 2.72, *p* < 0.05), and child vaccination (AOR = 0.15, *p* < 0.001).

**Conclusion:**

The prevalence of acute malnutrition among children aged 6-59 months was was higher than the national prevalence. The number of children in each household, drinking water sources, latrine availability, hand washing practice before food preparation and child feeding, childhood diarrheal disease, and child vaccination were identified as factors affecting the childhood acute malnutrition in the nomadic community. Protecting drinking water sources from possible contaminants, improving hand washing practices, utilization of latrine, preventing diarrheal diseases and vaccinating children integrated with the access of nutrition education is important to improve nutrition of children of the nomadic people.

## Background

Under nutrition continues to be a major health burden in the world, particularly in the developing world [1–3]. Globally, children with moderate and severe acute under nutrition are approximately 60 million and 13 million respectively [1, 2]. Under nutrition is globally the most important risk factor for illness and death, with hundreds of millions of young children particularly affected [4, 5]. It is associated with more than one third of the global disease burden for children [6]. Between 8 to 11 million under-five children die each year globally [2, 7], and more than 35% of these deaths are attributed to under nutrition [3].

Under nutrition among children is a critical problem. Its effects are long lasting [1, 8]. Under nourished children are physically, emotionally and intellectually less productive and suffer more from chronic illnesses and disabilities [9–12]. Malnutrition affects child performance, health, and survival [13, 14]. In the long term, early nutritional deficits are linked to impairments of intellectual performance; work capacity, reproductive outcomes and overall health during adolescence and adulthood [14–18]. The immediate consequences of poor nutrition during the early years include significant morbidity and mortality and delayed mental and motor developments. Malnutrition at the early stages of life can lower child resistance to infections [19]. Moreover, the potential negative impact of child malnutrition goes beyond the individual, affecting society and future generations [20, 21].

In Ethiopia, under nutrition among children is still a common problem. Ethiopia is one of the countries with very high burden of under nutrition. In the country, under nutrition is the underlying cause of 57% of child deaths [22–25].

Under nutrition among children depends on complex interactions of various factors, like socio-demographic [24–30], drinking water quality [26, 28, 29, 31–35], hygiene of complementary foods [24, 28], environmental sanitation [26, 31, 32, 36–38], child co-morbidities [19, 39–44], and child vaccination [45–47]. Though, the nature, magnitude and determinants of under nutrition are determined among the general populations, there is lack of evidence in the nomadic communities. This cross-sectional study was therefore conducted to assess the magnitude and factors associated with acute malnutrition among children aged 6–59 months in Hadaleala district, Afar Region, northeast Ethiopia.

## Methods

### Study design and settings

A community - based cross-sectional study was conducted among the nomadic populations in Hadaleala district, Afar Region, northeast Ethiopia in May, 2015. Hadaleala district is located at 341 km southwest of the regional capital, Semera, and 268 km north of Addis Ababa, the capital city of Ethiopia. It has an area of 1272 km^2^ divided into 11 rural kebeles (the smallest administrative units in Ethiopia) with a total population of 42,845 as projected for the year 2015. It has 7516 households with an average household size of 5.7 persons per house. Under-five children account for 10.1% (4328) of the total population. As the population lives in a very scattered manner, the average population density is 14 persons/km^2^_._ Furthermore, the economy of the district is based on livestock and crop production [48].

### Sample size determination

The sample size was determined using the single population proportion formula by considering the following assumptions: *p* = 10.0% (prevalence of malnutrition among children aged 6–59 months in Bule Hora district, South Ethiopia [49]), 95% confidence interval, and a 4% margin of error (d),

$$ n=\frac{{\left({z}_{\raisebox{1ex}{$\alpha $}\!\left/ \!\raisebox{-1ex}{$2$}\right.}\right)}^2p\left(1-p\right)}{d^2}=\frac{(1.96)^20.1\left(1-0.1\right)}{0.04^2}=217 $$

Considering the design effect of 2 and 10% non response rate, the final sample size was 478 mother-child pair.

### Sampling procedure

The multistage cluster sampling technique was used to select study participants from the nomadic population. The clusters were villages with defined geographical boundaries. Out of a total of 11 kebeles, 6 were selected by the simple random sampling technique. The 6 selected kebeles were clustered into 39 villages, and 17 villages were selected by the systematic random sampling technique. All the households (591) found in the selected 17 villages with children aged 6–59 months were included in the study. For households which had more than one child each, the younger one was selected for the study.

### Data collection tools and procedures

A structured questionnaire and anthropometric measurement were used to collect data. The questionnaire was pre-tested out of the study area in a community which had similar characteristics prior to the actual data collection. Eight diploma graduate nurses and two environmental health officers who were fluent enough in both Amharic and Afarigna (local languages) and working in the district were involved in the data collection process. Training was given for the data collectors and supervisors. The data collectors visited all households in the selected clusters. When the data collectors found the target groups during the visits, they interviewed the mothers about the variables and measured the circumference of the upper arm of the child. Finally, the collected data were checked and corrected by the data collectors immediately after finalizing the questionnaire. Supervisors daily checked the completeness, quality, and consistency of information collected.

### Measurement of outcome variable

Childhood malnutrition, the primary outcome variable of this study, is determined by a simple anthropometric index the so called mid-upper arm circumference (MUAC). Nutritional status of children was take as acute malnutrition if MUAC value is lower than 125 mm [50].

Childhood diarrheal disease, one of the predictor variables is defined as having three or more loose or watery stools in 24 h [51, 52]. Household economic status, which was the other predictor variable was calculated by using tropical livestock unit (TLU). Tropical livestock unit was determined by multiplying the number of specific species with the TLU conversion factor assigned to that specific species. Camels, cattle, sheep, goats, horses, mules, asses, and chickens were common in the study area. Generally, TLU was determined as (1.0_*_Number of camels) + (0.8_*_Number of horses) + (0.7_*_ Number of mules) + (0.7_*_Number of cattle) + (0.5_*_Number of asses) + (0.1_*_Number of sheep) + (0.1_*_Number of goats) + (0.01_*_Number of chickens). Household economic status was determined by comparing the TLU scores with the standard score. A below 5 TLU score indicated that the household was poor. A TLU score of 5 to 12.99 showed the household was medium in economic status, and rich households scored 13 and above TLU [53].

### Data management and statistical analysis

Data were entered using the EPI-INFO version 3.5.3 statistical package and exported to SPSS version 20 for further analysis. Cross tabulation was used to describe socioeconomic, environmental sanitation, health, and nutritional characteristics of children. Categorical data were presented as frequency counts or percentages and compared using the Pearson chi-square. Continuous data were summarized as mean or median with ± standard deviation and interquartile range. The univariable binary logistic regression analysis was used to choose variables for the multivariable binary logistic regression analysis, and variables which had less than 0.2 p – values by the univariable analysis were then analyzed by the multivariable binary logistic regression for controlling the possible effects of confounders, and finally, variables which had significant association were identified on the basis of adjusted odds ratio (AOR) with 95% CI and *p* < 0.05.

## Results

### Socio-demographic information

A total of 591 mothers - child pair participated in this study with a 100% response rate. More than half, 311 (52.6%) of the mothers were aged 25-34 years. The median age of the mothers was 30 years, and the interquartile range was 25-35 years. Almost all, 577 (97.6%) of the mothers were married at the time of data collection. The great majority, 514 (87.0%) of mothers were illiterate. Almost all, 559 (94.6%) of the mothers were housewives by occupation. Five hundred thirty – seven (90.9%) mothers were Afar by ethnicity. More than half, 339 (57.4%) of the households had more than five family members. Three hundred eighty – two (64.6%) households were economically poor. Two hundred twenty – nine (38.7%) of the children were aged above 35 months. The median age of children was 28 months and the interquartile range (IQR) was 16-40 months. More than half, 338 (57.2%) of the households had only one child aged 6-59 months, and 317 (53.6%) of the children were male (Table 1).

**Table 1:** Socioeconomic information of households (*n* = 591) in Hadaleala district, Afar region, northeast Ethiopia, April to May, 2015

Variables	Frequency	Percentage
Age of mothers in years		
15–24	131	22.2
25–34	311	52.6
≥ 35	149	25.2
Marital status of mothers		
Currently married	577	97.6
Currently not married	14	2.4
Educational level of mothers		
No formal education	514	87.0
Formal education	77	13.0
Occupational status of mothers		
Housewife	559	94.6
Employed	32	5.4
Ethnic group of mothers		
Afar	537	90.9
Oromo	44	7.4
Amhara	10	1.7
Family size		
≤ 5.	252	42.6
> 5	339	57.4
Household economic status		
Poor	382	64.6
Medium	209	35.4
Age group of children		
6.0–11.0	72	12.2
12.0–23.0	152	25.7
24.0–35.0	138	23.4
> 35.0	229	38.7
Sex of children		
Male	317	53.6
Female	274	46.4
Number of children		
One	338	57.2
Two	218	36.9
Three	35	5.9

### Drinking water and hygiene of complementary foods

Three hundred fifty – four (59.9%) households collected drinking water from unimproved sources and the greater majority, 522 (88.3%) of the water sources were seasonal. Very few, 20 (3.4%) households treated drinking water at home. Cow or goat milk was the commonest, 337 (57.0%) complementary food for the children. Three hundred thirty – three (56.3%) households served uncooked foods for the children, and the greater majority, 551 (93.2%) of the households used unclean utensils to serve foods. Three – forth, 447 (75.6%) of the households fed the children soon after the food is prepared, and the overwhelming majority, 539 (91.2%) used leftover foods. Three hundred twenty - four (54.8%) mothers washed hands with only water (Table 2).

**Table 2:** Drinking water and hygiene of complementary foods of households in Hadaleala district, Afar region, northeast Ethiopia, April to May, 2015

Environmental variables	Frequency	Percentage
Drinking water sources		
Improved	237	40.1
Unimproved	354	59.9
Seasonality of water sources		
Permanent	69	11.7
Temporarily	522	88.3
Home based water treatment		
Yes	20	3.4
No	571	96.6
Types of complementary foods for the children		
Cow or goat milk	337	57.0
Adults’ food	133	22.5
Gruel	112	19.0
Infant formula/Powder milk	9	1.5
Using unclean utensils to serve foods		
Yes	551	93.2
No	40	6.8
Serving uncooked food for the children		
Yes	333	56.3
No	258	43.7
Feeding children soon after food prepared		
Yes	447	75.6
No	144	24.4
Children ate leftover foods		
Yes	539	91.2
No	52	8.8
How do you wash your hand		
With plain water	324	54.8
With soap	267	45.2

### Personal hygiene and environmental sanitation

Nearly one – tenth, 56 (9.5%) of the mothers had good personal hygiene. More than threefold, 483 (81.7%), and 490 (82.9%) of the households practiced open defecation and indiscriminate solid waste disposal respectively. The living environment of 464 (78.5%) households was poor condition, and vector infestation was observed among 483 (81.7%) households. Three hundred thirty (55.8%) households had only one room, and very few, 91 (15.4%) households had cemented or plastered floor (Table 3).

**Table 3:** Personal hygiene and environmental sanitation of households in Hadaleala district, Afar region, northeast Ethiopia, April to May, 2015

Environmental variables	Frequency	Percentages
Personal hygiene of mothers		
Poor	535	90.5
Good	56	9.5
Latrine availability		
Yes	108	18.3
No	483	81.7
Solid waste management		
Controlled	101	17.1
Open field	490	82.9
Environmental sanitation		
Poor	464	78.5
Good	127	21.5
Infestation of insects		
Yes	483	81.7
No	108	18.3
Number of rooms		
One	330	55.8
Two	231	39.1
Three	30	5.1
Housing floor material		
Earth/sand	500	84.6
Cemented	91	15.4

### Health condition of mothers and children

Sixty (10.2%) mothers and 172 (29.1%) children had diarrheal disease in the 2 week period prior to the survey. A majority, 416 (70.4%) of the mothers didn’t know the causes of diarrhea. Three hundred twenty – seven (55.3%) and 377 (63.8%) mothers didn’t know that flies and child excreta can cause diarrheal diseases, respectively. The great majority, 477 (80.7%) of the children had ever been vaccinated. However, significant number or proportion, 254 (43.0%) and 418 (70.7%) of the children had no measles and rotavirus vaccination respectively. Four hundred eighty- eight (82.6%) of the children received vitamin A supplementation (Table 4).

**Table 4:** Health conditions of mothers and children in Hadaleala district, Afar region, northeast Ethiopia, April to May, 2015

Health related information	Frequency	Percentage
Two week history of maternal diarrhea		
Yes	60	10.2
No	531	89.8
Childhood diarrhea		
Yes	172	29.1
No	419	70.9
Mothers know the causes of diarrheal disease		
Yes	175	29.6
No	416	70.4
Mothers know flies transmit diarrheal disease		
Yes	264	44.7
No	327	55.3
Mothers know excreta of children can cause disease		
Yes	214	36.2
No	377	63.8
Child ever been vaccinated		
Yes	477	80.7
No	114	19.3
Measles vaccination		
Yes	337	57.0
No	254	43.0
Rota virus vaccination		
Yes	173	29.3
No	418	70.7
Vitamin A supplementation		
Yes	488	82.6
No	103	17.4

### Nutritional status

**Fig. 1:**
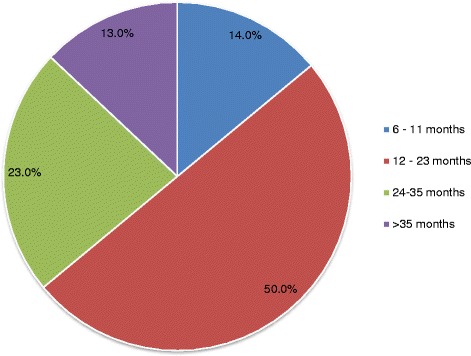
Prevalence of acute malnutrition with respect to age of children in Hadaleala district, Afar region, northeast Ethiopia, April to May, 2015

The MUAC value of 70 children was below 125 mm. Therefore, the prevalence of acute malnutrition among children aged 6 - 59 months in the nomadic population of Hadaleala district, Afar Region was found to be 11.8% (95% CI = 9.3, 14.8%). Female children were more malnourished than males. Out of 70 malnourished children, 42 (60%) females and 28 (40%) males were malnourished respectively. The highest prevalence of acute malnutrition occurred among children aged 12-23 months, which accounted 35 (50%) (Fig. 1).

### Factors associated with nutritional status

Table 5 presents the results of the binary logistic regression analysis on socioeconomic, water and hygiene of complementary foods, personal hygiene and environmental sanitation, and health related variables. Childhood acute malnutrition was statistically associated with the number of children in the household. Acute malnutrition was 2.49 times more likely to be higher among households with two children compared with households with only one child [AOR = 2.49, 95% CI = (1.06, 5.85)]. Similarly, the likelihood of acute malnutrition was also 12.87 times higher among households with three children compared with households who had one child [AOR = 12.87, 95% CI = (4.04, 41.00)].

**Table 5:** Factors affecting acute malnutrition among children aged between 6 and 59 months in Hadaleala district, Afar region, northeast Ethiopia, April to May, 2015

Variables	Acute malnutrition		COR with 95% CI	AOR with 95% CI
	Yes	No		
Number of children				
One	13	325	1	
Two	35	183	4.78 (2.47, 9.27)	2.49 (1.06, 5.85)*
Three	22	13	42.31 (17.52, 102.18)	12.87 (4.04, 41.00)**
Family size				
≤ 5	37	215	1	
> 5	33	306	0.63 (0.38, 1.03)	1.99 (0.95, 4.18)
Wealth status				
Poor	58	324	2.94 (1.54, 5.61)	1.68 (0.72, 3.94)
Medium	12	197	1	
Mothers’ occupation				
House wife	63	496	0.45 (0.19, 1.09)	0.20 (0.04, 1.05)
Employee	7	25	1	
Water sources				
Protected	4	233	1	
Unprotected	66	288	13.35 (4.80, 37.16)	3.78 (1.07, 13.34)*
Latrine availability				
Yes	4	104	1	
No	66	417	6.61 (2.04, 21.40)	5.24 (1.19, 23.19)*
Hand washing** *				
With water only	66	258	1	
With soap	4	263	0.06 (0.02, 0.17)	0.21 (0.05, 0.81)*
Children eat leftover foods				
Yes	61	478	0.61 (0.28, 1.31)	0.68 (0.23, 2.01)
No	9	43	1	
Childhood diarrhea				
No	11	408	1	
Yes	59	113	19.37 (9.85, 38.10)	2.72 (1.15, 6.40)*
Children ever vaccinated				
Yes	19	458	0.05 (0.03, 0.09)	0.15 (0.07, 0.31)**
No	51	63	1	
Solid waste management				
Controlled	15	62	2.02 (1.08, 3.79)	1.89 (0.62, 5.75)
Uncontrolled	55	459	1	

*Statistically significant at *p* < 0.05 | **statistically significant at *p* < 0.001 | ***before food preparation and child feeding

Acute malnutrition among children aged 6 - 59 months was associated with drinking water sources, availability of latrine, and hand washing practices. It was 3.78 times more likely to be higher among households that collected drinking water from unprotected sources [AOR = 3.78, 95% CI = (1.07, 13.34)]. The likelihood of childhood acute malnutrition was 5.24 times to be higher among households who had no latrine compared with their counterparts [AOR = 5.24, 95% CI = (1.19, 23.19)]. Children whose mothers washed their hands before food preparation and feeding with soap were less likely to be malnourished. Hand washing with soap before food preparation and child feeding can prevent childhood acute malnutrition by 79% [AOR = 0.21, 95% CI = (0.05, 0.81)].

Childhood acute malnutrition was also statistically associated with the health status of children, like childhood diarrhea and vaccination. Childhood acute malnutrition was 2.72 times more likely to be higher among children who had diarrheal disease [AOR = 2.72, 95% CI = (1.15, 6.40)]. This study indicated that child vaccination has a protective effect on childhood acute malnutrition. Children who ever been vaccinated were 85% less likely to be malnourished, compared with their counterparts [AOR = 0.15, 95% CI = (0.07, 0.31)].

## Discussion

The prevalence of acute malnutrition among children aged 6-59 months was 11.8% (95% CI = 9.3, 14.8%). Childhood acute malnutrition was statistically associated with the number of children in each household, drinking water sources, latrine availability, hand washing practice before food preparation and child feeding, childhood diarrheal disease, and child vaccination. The prevalence of acute malnutrition reported by this study is slightly higher than the national prevalence of acute malnutrion (9%) [54] and findings of various studies conducted in Ethiopia like Bule Hora district, South Ethiopia, 10% [49] and it was also just two-fold higher than the prevalence reported in Aleta Chucko and Aleta Wondo districts, Sidama Zone, South Ethiopia, 5.6% [55]. Whereas, the magnitude of acute malnutrition reported by this study was lower than the findings of studies conducted in Pagak district, South Sudan, 16.7% [56]. The difference in prevalence might be attributed to the difference in the socio- demographic, environmental, and behavioral characteristics of households and the nomadic nature of the population.

This study showed that families who had two or above children aged 6-59 months were more likely to have childhood acute malnutrition than those who had only one child. This probably attributed to less balanced diet intake and accessibility of child healthcare decreased with more number of children per household, especially in low income families [33, 34, 57, 58].

In this study, it was found that acute malnutrition was associated with unprotected drinking water sources, open defecation, and poor hand washing practices of mothers. Different studies also reported that acute malnutrition was associated with drinking water sources [26, 28, 29, 31–35], availability of latrine [26, 31, 32, 36–38] and hand washing practices [36, 59]. This may be so because poor water, hygiene and sanitation condition increase the risk of infections. Infections affect nutrient absorption and compromised nutritional status of children. Evidences show that children who frequently affected by infections have mal-absorption of important nutrients [60–63].

This study indicated that acute malnutrition was associated with child hood diarrheal diseases. Children who had diarrheal disease were more likely to be acutely malnourished as compared with their counter parts. This finding was supported by the findings of other similar studies [24, 28, 33, 36, 38, 64–66]. This may be due to the fact that diarrheal disease due to poor hygiene and lack of sanitation induces a gut disorder called environmental enteropathy (EE) characterized by blunted intestinal villi, increased intestinal permeability; fat and carbohydrate mal-absorption, and increased protein needs [67] that diverts energy from growth towards an ongoing fight against subclinical infection [68–71]. EE is a major cause of post-natal stunting and wasting [71–77].

Child vaccination was also the other statistically associated variable with childhood acute malnutrition. Children who ever vaccinated were less likely to be malnourished compared with their counterparts. This finding is supported by the findings of other similar studies [36, 45–47, 78]. This can be justified as vaccinated children are less likely to be frequently infected with vaccine preventable diseases such as diarrhea and respiratory infections, which are known in depleting nutrients from the body [45, 79, 80].

Finally, this paper determined acute malnutrition using MUAC measurement. It didn’t measure weight and height to determine global malnutrition. This paper also didn’t consider the effect of food security and access to diversified foods on childhood malnutrition. Moreover, the paper didn’t investigate demand side issues and supply side issues of systems failures with respect to poverty alleviation. The authors believed that other studies should be conducted to fill the above identified gaps.

## Conclusion

The prevalence of acute malnutrition among children aged 6-59 months was higher than the national prevalence. The number of children in each household, drinking water sources, latrine availability, hand washing practice before food preparation and child feeding, childhood diarrheal disease, and child vaccination were identified as factors affecting the childhood acute malnutrition in the nomadic community. Protecting drinking water sources from possible contaminants, improving hand washing practices, utilization of latrine, preventing diarrheal diseases and vaccinating children integrated with the access of nutrition education is important to improve nutrition of children of the nomadic people.

## Abbreviations

AOR: Adjusted Odds Ratio
CI: Confidence Interval
COR: Crude Odds Ratio
EE: Environmental Enteropathy
IQR: Interquartile Range
Km: Kilometer
Km^2^: Kilometer square
MUAC: Mid-upper arm circumference
SPSS: Statistical Package for Social Sciences
TLU: Tropical Livestock Holding
